# Recent Advances in Immunotherapy for Advanced Biliary Tract Cancer

**DOI:** 10.1007/s11864-024-01243-y

**Published:** 2024-07-27

**Authors:** Shiwei Yue, Yunpu Zhang, Wei Zhang

**Affiliations:** 1 Hepatic Surgery Center, Tongji Hospital, Tongji Medical College, Huazhong University of Science and Technology, 1095 Jiefang Avenue, 430030 Wuhan, China; 2 Hubei Key Laboratory of Hepato‑Pancreatic‑Biliary Diseases, Tongji Hospital, Tongji Medical College, Huazhong University of Science and Technology, 1095 Jiefang Avenue, 430030 Wuhan, China; 3 Clinical Medical Research Center of Hepatic Surgery at Hubei Province, 1095 Jiefang Avenue, 430030 Wuhan, China

**Keywords:** Biliary tract cancer, Immunotherapy, Checkpoint inhibitors, Cancer vaccines, Adoptive cell therapy

## Abstract

Biliary tract cancer (BTC) is a heterogeneous group of aggressive malignancies that arise from the epithelium of the biliary tract. Most patients present with locally advanced or metastatic disease at the time of diagnosis. For patients with unresectable BTC, the survival advantage provided by systemic chemotherapy was limited. Over the last decade, immunotherapy has significantly improved the therapeutic landscape of solid tumors. There is an increasing number of studies evaluating the application of immunotherapy in BTC, including immune checkpoint inhibitors (ICIs), cancer vaccines and adoptive cell therapy. The limited response to ICIs monotherapy in unselected patients prompted investigators to explore different combination therapy strategies. Early clinical trials of therapeutic cancer vaccination and adoptive cell therapy have shown encouraging clinical results. However, there still has been a long way to go via validation of therapeutic efficacy and exploration of strategies to increase the efficacy. Identifying biomarkers that predict the response to immunotherapy will allow a more accurate selection of candidates. This review will provide an up-to-date overview of the current clinical data on the role of immunotherapy, summarize the promising biomarkers predictive of the response to ICIs and discuss the perspective for future research direction of immunotherapy in advanced BTC.

## Introduction

Biliary tract cancer (BTC) is a heterogeneous group of aggressive malignancies that arise from the epithelium of the biliary tract [1]. Based on the anatomical location, BTC is classified as gallbladder carcinoma (GBC), intrahepatic cholangiocarcinoma (iCCA) and extrahepatic cholangiocarcinoma (eCCA). The latter is further subdivided into perihilar cholangiocarcinoma (pCCA or Klatskin tumor) and distal cholangiocarcinoma (dCCA) [2]. Due to the differences in geographic distribution and ethnic factors, the incidence of cholangiocarcinoma (CCA) is low (between 0.35 and 2 cases per 1,000,000 annually) in high-income countries. However, it is up to 40 times higher in endemic regions, including Thailand and China. There has been a steady increase in the incidence of BTC in recent decades, mainly from iCCA but also from eCCA. The incidence of GBC is relatively stable or decreasing in high-income countries, which is probably attributed to the increase in routine cholecystectomy [3]. The main established risk factors associated with BTC include primary sclerosing cholangitis, liver fluke infections, hepatolithiasis, Caroli’s disease, cirrhosis, chronic viral infections (hepatitis virus B and hepatitis virus C), obesity and nonalcoholic fatty liver disease [4]. Notably, most patients with BTC have no identifiable risk factors. Despite variations in pathophysiology, GBC and CCA are often classified together and treated similarly, particularly in advanced stages.

Surgical resection remains the only current potentially curative treatment option for patients with early-stage BTC. However, most patients present with locally advanced or metastatic disease at the time of diagnosis due to the absence of early specific symptoms. Curative surgical resection with negative tumor margins can be achieved in less than 30% of patients [5]. Moreover, the risk of locoregional and distant relapse is still high even after radical resection. The 5-year disease-free survival (DFS) can be as low as 20% depending on disease stage [6]. In selected patients with localized and unresectable disease, locoregional therapies, including transarterial chemoembolization, hepatic arterial chemotherapy infusion, radioembolization, radiofrequency ablation, and radiotherapy, can be considered [7]. Unfortunately, none have been validated in prospective randomized controlled trials. Therefore, systemic therapy is currently the mainstay of palliative therapy for patients with advanced or recurrent BTC. Based on the results of the landmark phase III ABC-02 study, the combination of gemcitabine and cisplatin represents the standard first-line treatment for patients with locally advanced or metastatic disease, although median overall survival remains poor at less than 1 year [8]. In the phase III ABC-06 trial, second-line therapy with modified 5-fluorouracil/folinic acid/oxaliplatin (FOLFOX) offered a modest overall survival (OS) improvement (median OS, 6.2 vs. 5.3 months) compared to active symptom control alone [9]. The overall limited survival advantage provided by systemic chemotherapy emphasizes the unmet need for more effective treatments in advanced BTC.

The recent advent of molecular testing and next-generation sequencing has led to the delineation of the genetic landscape of BTC, suggesting substantial molecular heterogeneity across iCCA, eCCA, and GBC. Several clinical trials have evaluated the efficacy of novel targeted agents against BTC with certain molecular alterations. In the ClarIDHy phase III trial, patients with isocitrate dehydrogenase (IDH1)-mutated CCA who progressed on previous therapies responded considerably to ivosidenib in terms of both progression-free survival (PFS) and OS [10]. In the FIGHT-202 trial, pemigatinib was found to have higher response rates (36%, including three complete responses) and disease control rates (DCR) (80%) in patients with CCA and fibroblast growth factor receptor (FGFR)-2 fusions or rearrangements than in patients without FGF/FGFR alterations, with a duration of disease control of approximately 7.5 months [11]. The neurotrophic tyrosine receptor kinase (NTRK) inhibitors larotrectinib and entrectinib have shown high response rates (75%) and durable responses in early-phase trials of NTRK fusion-positive advanced solid tumors, including CCA [12]. Both have been approved by the US FDA for patients with NTRK fusion-positive tumors.

In the last decade, immunotherapy has significantly improved the therapeutic landscape of solid tumors, including melanoma [13], lung cancers [14], renal cancers [15], and hepatocellular carcinoma [16]. There is an increasing number of studies evaluating the application of immunotherapy in BTC, including immune checkpoint inhibitors (ICIs) targeting programmed death 1 (PD-1), programmed death-ligand 1 (PD-L1), and cytotoxic T lymphocyte antigen-4 (CTLA-4), cancer vaccines and adoptive cell therapy [17]. Although these new treatment strategies have improved patient survival, the effectiveness of ICI monotherapy remains relatively limited, with an objective response rate (ORR) of up to approximately 20% [18]. Numerous trials are being conducted to investigate therapeutic strategies using combinations of ICIs with immunotherapy, chemotherapy, antiangiogenics, PARP inhibitors and local therapy, with preliminary results showing promising antitumor efficacy and tolerable safety [19]. Notably, only a minority of patients with advanced BTC are sensitive to immunotherapy. The mechanisms underlying the disparities in the response to immunotherapy are poorly defined. Therefore, the identification of predictive biomarkers for immunotherapy has become urgently warranted.

**Fig.1:**
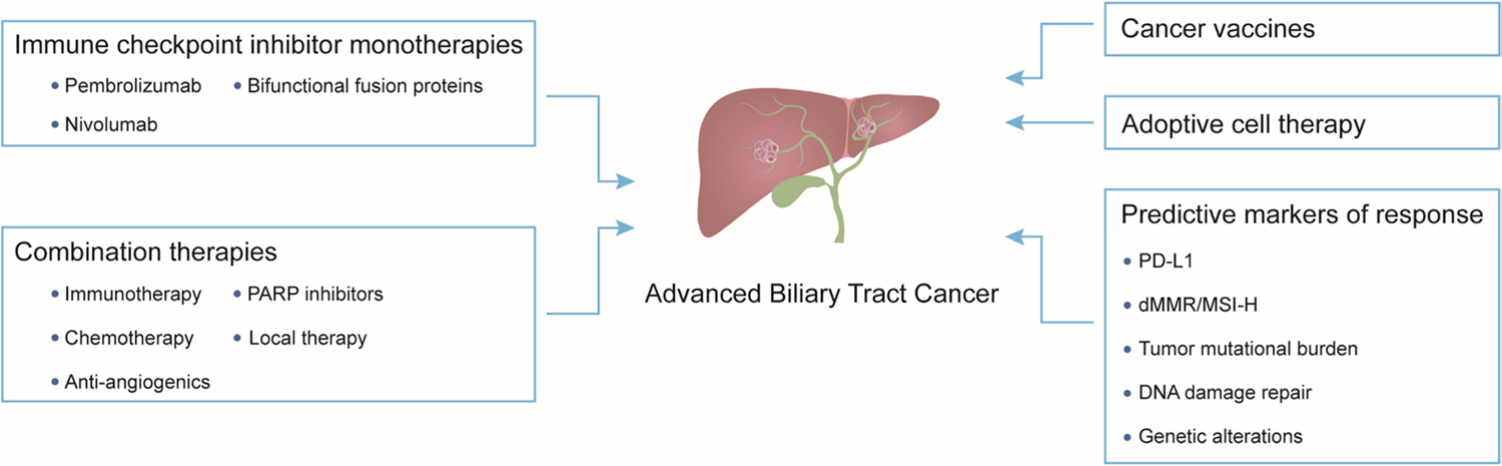
Graphic summary of recent advances in immunotherapy for advanced biliary tract cancer

In this review, we provide an up-to-date overview of the current clinical data on the role of immunotherapy in advanced BTC. Furthermore, we summarize promising biomarkers that could serve as predictors of the response to immunotherapy and discuss perspectives for future research directions of immunotherapy in advanced BTC (Fig. 1).

## Immune Checkpoint Inhibitor (ICI) Monotherapies

### PD-1 Inhibitor: Pembrolizumab

Multiple small studies have evaluated the efficacy of immune checkpoint inhibitors as monotherapy in BTC. Pembrolizumab, a highly selective, humanized monoclonal antibody that binds to the PD-1 receptor and blocks its interaction with its ligands, PD-L1 and programmed death ligand 2, has demonstrated activity as monotherapy in numerous tumor types and hematologic malignancies.

At the European Society for Medical Oncology (ESMO) Asia 2015 Congress, the interim results from KEYNOTE-028, a phase Ib study of pembrolizumab in patients with advanced solid tumors, were reported [20]. Twenty-four BTC patients with PD-L1 expression experienced an ORR and DCR of 17% and 34%, respectively. Data from KEYNOTE-028 based on approximately 2 years of additional follow-up have been recently published [21]. After a median follow-up of 5.7 months (0.6–55.4), the ORR was 13%, and the median OS and PFS were 5.7 (3.1–9.8) and 1.8 months (1.4–3.1), respectively. A total of 16.7% of patients experienced grade 3 treatment-related adverse events (AEs). There were no grade 4 to 5 treatment-related AEs. Lee et al. [22] evaluated the efficacy of pembrolizumab in patients with advanced MMR-deficient cancers across 12 different tumor types in 2017. Among four CCA patients, three achieved stable disease (SD), and one achieved complete response (CR). Thus, the DCR was 100%. In May 2017, the U.S. Food and Drug Administration granted accelerated approval to pembrolizumab for patients with unresectable or metastatic, MSI-H or dMMR solid tumors. In June 2020, pembrolizumab was approved by the FDA for TMB-high (more than 10 mutations per megabase) noncolorectal malignancies. To date, KEYNOTE-158 (NCT02628067) is the largest study of ICI monotherapy with pembrolizumab [23]. Among 104 patients with advanced biliary cancers after progression or intolerance to standard therapy, the confirmed ORR was 5.8%; the median duration of response (DOR) was not reached (6.2–26.6 months). The median OS and PFS were 7.4 and 2.0 months, respectively. The rate of grade 3 to 5 treatment-related AEs was 13.5%. Among 22 patients with MSI-H/dMMR CCA who were enrolled in the same study, the ORR was 40.9%. The median OS and PFS were 24.3 and 4.2 months, respectively [24]. Therefore, pembrolizumab monotherapy provided durable antitumor activity, regardless of PD-L1 expression, and manageable toxicity.

### PD-1 Inhibitor: Nivolumab

The anticancer activity of the human immunoglobulin G4 monoclonal antibody nivolumab was evaluated in 54 patients with histologically confirmed unresectable or metastatic CCA or gallbladder cancers who were refractory or intolerant to at least 1 line of systemic therapy but no more than 3 prior lines of systemic therapy [25]. Among 46 examined patients who had tumor response evaluation with radiologic imaging, investigator-assessed PR and SD were achieved by 10 patients (22%) and 17 patients (32%), respectively, and thus, the consequent DCR was 59%. Four responders (40%) achieved a durable objective response lasting at least 1 year. mPFS was 3.68 months, and mOS was 14.24 months. The National Cancer Institute Molecular Analysis for Therapy Choice (NCI-MATCH) trial enrolled three patients with MMR-deficient CCA treated with nivolumab [26]. CR was observed in 1 patient with CCA. The other two patients had PD as the best response.

### Bifunctional Fusion Proteins

Higher expression of transforming growth factor-beta (TGF-β) is associated with immune escape, resistance to current cancer therapy, and poor prognosis in advanced tumors, including biliary tract cancer [27]. While anti-TGF-β therapeutics offer a potential treatment for cancer, these agents have had little effectiveness in the clinic as monotherapies thus far [28]. M7824 (MSB0011359C) is a first-in-class bifunctional fusion protein composed of the extracellular domain of two TGF-β receptor 2 molecules fused to a fully humanized monoclonal antibody against PD-L1. M7824 simultaneously blocks the PD-L1 and TGF-β pathways of immune evasion, having an impact on both the innate and adaptive immune systems [29]. Thus, M7824 demonstrates encouraging clinical activity and a manageable safety profile in patients with pretreated advanced solid tumors [30].

The data from a phase I trial demonstrated the clinical activity of M7824 in 30 Asian patients with BTC whose disease progressed after standard chemotherapy. The ORR was 20%, and the total clinical response was 30%. The median PFS and OS were 2.5 months and 12.7 months, respectively. Eleven (37%) patients developed grade ≥ 3 TRAEs, including 3 patients who died due to septic shock and interstitial lung disease [31]. Based on these findings, further study of M7824 treatment in patients with BTC is warranted; for example, its use as a monotherapy in second-line BTC (NCT03833661), in combination with gemcitabine and cisplatin (NCT04066491), and in combination with hypofractionated radiation therapy (NCT04708067) is worthy of further exploration.

## Combination Therapies

### ICI In Combination with Immunotherapy

The limited antitumor effect of immune checkpoint inhibitor monotherapy in advanced CCA has prompted investigators to explore different combination therapy strategies. Combining CTLA-4 and PD-1 blockers may have a synergistic effect on activating the antitumor immune response [32]. The results of clinical trials evaluating the effectiveness of CTLA-4 + PD-1 blockers showed that combination therapy was beneficial for metastatic melanoma, renal cell carcinoma, and lung cancer [33–35].

Durvalumab is being investigated in phase I studies in Asian patients with biliary tract cancer [36]. The results showed the superiority of the durvalumab–tremelimumab combination versus durvalumab alone in terms of OS (10.1 vs. 8.1 months) and DCR at 12 weeks (32.2 vs. 16.7%). The median DORs for durvalumab and the durvalumab–tremelimumab combination were 9.7 and 8.5 months, respectively. No unexpected toxicities were observed. The CA209-538 trial is a multicenter, nonrandomized open label phase 2 study conducted in Australia for patients with rare advanced cancers after one or more lines of therapy [37]. In this trial, patients received treatment with nivolumab and ipilimumab. The ORR in the BTC subgroup was 23%, with a DCR of 44%. Of note, responses were limited to patients with ICC and GBC, suggesting that the response to dual checkpoint inhibitor therapy in BTC may differ by anatomical site. The median PFS was 2.9 months, and the OS was 5.7 months. Immune-related grade 3 or 4 events were reported in 15% of patients. Tumor-associated macrophages (TAMs) make up the majority of immunosuppressive cells in the tumor microenvironment in CCA. The myelopoiesis, recruitment and polarization of TAMs are mediated by tumor-derived granulocyte–macrophage colony-stimulating factor (GM-CSF) [38]. Blocking the GM-CSF axis enhances antitumor T-cell immunity [39]. In a phase II trial, Kelley et al. investigated the efficacy and safety of pembrolizumab in combination with GM-CSF in 27 advanced BTC patients whose disease worsened after at least one prior therapy [40]. Five of the 24 patients (21%) achieved partial responses. Progression-free survival at 6 months was 35%.

### ICI In Combination with Chemotherapy

Chemotherapy has been shown to have low therapeutic efficacy in clinical trials due in part to the immunosuppressive nature of tumor microenvironments. Recent findings have demonstrated a direct or indirect immunostimulatory effect of chemotherapeutic drugs [41]. There is growing evidence that chemotherapy may improve the effectiveness of immunotherapy by reducing the immunosuppressive effects in the microenvironment, increasing the cross-presentation of tumor antigens, and facilitating immune cell infiltration into the tumor core [42].

Nivolumab as monotherapy or in combination with cisplatin plus gemcitabine chemotherapy has been evaluated as a first-line treatment in Japanese patients with advanced BTC [43]. The median OS and PFS for the monotherapy cohort were 5.2 months and 1.4 months, respectively. One of 30 patients (3%) had an objective response. The ORR was 37% in the combination cohort, with an mPFS of 4.2 months and an mOS of 15.4 months. A multi-institutional phase 2 clinical trial (BilT-01) evaluated the efficacy and safety of nivolumab in combination with gemcitabine and cisplatin or ipilimumab in patients with advanced BTCs and no prior systemic therapy [44]. The median PFS and OS in the chemo-IO combination arm were 6.6 and 10.6 months and in the IO-IO combination arm were 3.9 and 8.2 months, respectively. Of interest, the observed OS rate at 2 years was 35.4% in the chemo-IO combination arm. However, the probability of survival with chemotherapy alone at 2 years is estimated to be 15%-22% [45, 46]. Gemcitabine and cisplatin plus durvalumab for patients with advanced BTC have been evaluated in the phase 3 TOPAZ-1 study [47]. A total of 685 patients with inoperable, locally advanced, recurrent, or metastatic BTC were randomly assigned to receive durvalumab or a placebo. OS and PFS were significantly improved in the durvalumab group. The ORR was 26.7% for durvalumab and 18.7% for placebo. TOPAZ-1 is the first phase 3 trial to show that adding immunotherapy to standard chemotherapy can increase survival in biliary tract cancer without inducing any new serious side effects. Boilève A et al. [48] reported the safety run-in results of the triplet combination of durvalumab, tremelimumab, and paclitaxel in patients with advanced BTC in a noncomparative randomized phase II study (IMMUNOBIL PRODIGE 57). Among 20 enrolled patients, five patients in the triplet combination arm had dose-limiting toxicities (DLTs), meeting a stopping rule for the trial inclusions.

In combination with chemotherapy, other anti-PD-1 drugs (mostly camrelizumab and toripalimab) have demonstrated some efficacy in advanced BTC. Toripalimab in combination with gemcitabine and S-1 (GS) showed encouraging efficacy and safety in a single-arm, phase II trial including 50 patients with advanced BTCs. The median PFS was 7 months, and the median OS was 15 months. The disease control rate was 87.8%, and the ORR was 30.6% [49]. The combination of camrelizumab and a gemcitabine and oxaliplatin (GEMOX) regimen in 54 advanced BTC patients was tested in a phase II trial [50]. The 6-month PFS rate was 50%. Twenty (54%) out of 37 patients had an objective response, with a DCR of 89%. The median PFS and OS were 6.1 months and 11.8 months, respectively.

Currently, several trials are evaluating various combination approaches, including cytotoxic chemotherapy with ICIs [51]. Moreover, the results from the phase 3 KEYNOTE-966 trial (gemcitabine and cisplatin plus pembrolizumab or placebo; NCT04003636) and the phase 2 EORTC1607 trial (gemcitabine and cisplatin plus pembrolizumab; NCT03260712) are forthcoming.

### ICI In Combination with Anti-Angiogenics

In the vast majority of solid tumors, vascular endothelial growth factor (VEGF) is overexpressed and is commonly regarded as a primary mediator of tumor angiogenesis. There are intricate relationships between angiogenesis and immunity in tumors. VEGF inhibits immunological activation by inhibiting the development of dendritic cells [52]. In addition, VEGF could increase PD-1 expression on T cells and mediate CD8 T-cell exhaustion in malignancies [53]. Several immunostimulatory effects of antiangiogenic therapy have been identified, including an increase in the trafficking of T cells into tumors and a decrease in immunosuppressive cytokines and T regulatory cells [54, 55]. Initial clinical evidence also supports the synergistic interaction between antiangiogenic therapy and immunotherapy [56].

Arkenau HT et al. [57] reported the first phase I trial combining antiangiogenic agents (ramucirumab, an IgG1 VEGFR-2 antagonist) with pembrolizumab in patients with previously treated advanced BTC in 2018. Unfortunately, pembrolizumab plus ramucirumab had dismal clinical outcomes, with median PFS and OS of 1.6 months and 6.4 months, respectively. This combination therapy did not demonstrate a significant survival benefit compared with conventional treatment modalities. The combination of pembrolizumab or nivolumab with lenvatinib has shown remarkable results in a retrospective study involving 56 patients with advanced BTC, where the ORR and DCR reached 30.4% and 85.7%, respectively [58]. The median PFS and OS were 5 and 11 months, respectively. In addition, PD-L1-positive expression was associated with a better ORR and a longer PFS. Recently, an open-label phase II trial studied the efficacy of combining lenvatinib with a PD-1 inhibitor for the treatment of advanced BTC for the first time [59]. Thirty-eight patients were enrolled, with an ORR of 42.1% and DCR of 76.3%. The median OS was 17.7 months, and the 1-year OS rate was 47.4%. Thirteen (34.2%) patients achieved downstaging and underwent surgery, six of whom (46.2%) achieved a pathologic response. Treatment response and prognosis were related to the histopathological type of BTC. Patients with GBC showed a greater ORR (61.5%) than those with ICC (40%) or ECC (0%). For patients with advanced gallbladder cancer (GBC), this combination therapy has been shown to be an effective and tolerable therapy in a preliminary single-arm exploratory study [60]. Ten (32.3%) out of 31 patients achieved an objective response. The DCR was 83.9%, with a median PFS of 5.0 months and a median OS of 11.3 months. Encouragingly, three (9.7%) patients were reassigned to surgery, and one patient achieved a pathological complete response.

Currently, no standard second-line systemic treatment regimen has been recommended for advanced BTC. A real-world retrospective study in China included 74 patients with advanced BTCs who experienced progression after CisGem in first-line therapy [61]. Patients treated with lenvatinib plus a PD-1 inhibitor achieved an ORR of 20.27% and a DCR of 71.62%. The mPFS was 4.0 months, and the median OS was 9.5 months. A total of 52.7% of patients developed grade 3/4 AEs. Recently, Lulu X et al. [62] assessed the efficacy of lenvatinib with a PD-1 inhibitor in a single-arm retrospective study focusing on ICC patients after chemotherapy failure. The ORR was 17.5%, and the DCR was 75.0%. The median PFS and 6-month PFS rates were 5.8 months and 32.5%, respectively. The median OS was 14.3 months, and the 12-month and 18-month rates were 61.4% and 34.7%, respectively. Therefore, this combination could provide a new treatment option for advanced ICC.

A combination of angiogenesis/checkpoint blockade with chemotherapy may further enhance antitumor immune responses. In a phase II trial, the combination of toripalimab and lenvatinib plus GEMOX was evaluated as a first-line therapy for locally progressed or metastatic ICC [63]. The ORR was 80% (24/30), and the DCR was 93.3% (28/30) among the 30 enrolled patients. CR was observed in one patient. Three patients with locally advanced disease received resection after being successfully downstaged. The median PFS was 10.0 months, and the median DOR was 9.8 months.

### ICI In Combination with PARP Inhibitors

Accumulating evidence suggests that targeting the DNA damage response (DDR) has antitumor effects in a variety of cancer types. The PARP family of proteins plays critical roles in maintaining genomic stability and avoiding the accumulation of DNA damage [64]. PARP inhibitors are the only DDR-targeted agents approved by the FDA for a subset of patients with BRCA mutations and breast, ovarian, or pancreatic cancers [65]. As a tumor suppressor gene, BRCA encodes a nucleic acid protein that regulates transcription, double-stranded DNA repair, and DNA recombination [66]. The incidence of BRCA1/2 mutations in BTC patients varies between 1 and 7% [67, 68].

Xiong F et al. [69] described a patient with BRCA1-mutated and PD-L1-positive recurrent iCCA who received olaparib (a PARP inhibitor) and pembrolizumab. The patient achieved CR without significant adverse events. Such combinations have shown encouraging antitumor activity in other malignancies [70]. Multiple clinical trials are now evaluating the potential role of PARP inhibitors combined with ICIs in metastatic BTC. The combination of the PARP inhibitor olaparib and durvalumab is currently being evaluated in a phase II trial (NCT03991832) in patients with advanced IDH-mutated solid tumors, including CCA. The primary outcomes of this trial are ORR and DCR. The estimated enrollment is 58 patients, and the estimated primary completion date is March 2024. A multicenter phase II trial of rucaparib in combination with nivolumab as maintenance therapy for patients with advanced biliary tract cancer following platinum therapy was recruiting (NCT03639935), with an estimated primary completion date in April 2023.

### ICI In Combination with Local Therapy

Local tumor destruction combined with immunotherapy may have a synergistic effect against solid tumors [71]. In addition to reducing tumor burden, radiotherapy can trigger antitumor immunity and reprogram of the tumor microenvironment [72]. By directly destroying DNA, irradiation allows tumor cells to release more neoantigens, which activate the immune system. As a result of localized irradiation, chemokines are released, and T cells are be recruited, converting tumors into immune-susceptible tumors [73].

Reports on the efficacy of immunotherapy combined with radiotherapy in advanced CCA were mainly case reports and small case series. In a case of a chemotherapy-intolerable stage IV ICC patient with multiple distant metastases, a combination of anti-PD-1 immunotherapy and radiotherapy was adopted as first-line treatment [74]. After 26 months of treatment, combined therapy eventually resulted in a complete response to the primary tumor and all metastases. Zhao Q et al. [75] reported four cases of refractory advanced ICC or hilar CCA effectively managed with anti-PD-1 antibody treatment following or concomitant with stereotactic body radiotherapy (SBRT). The median OS of these patients was greater than 1 year. Radical surgical resection was performed in one patient with a large lesion (12.9 × 11.8 cm) and multiple satellite lesions who was initially deemed unresectable. The combination strategy exhibited good antitumor activity, sustained clinical efficacy, and a favorable safety profile. A retrospective report on the therapeutic responses of PD-1 blockade combined with SBRT revealed that late-stage or recurrent ICC patients can benefit from this combination therapy, even if they had low TMB, pMMR, MSS or negative PD-L1 expression [76]. It is worth noting that strong abscopal effects were observed in all three patients. Both the recurrent intrahepatic lesion and the lymph node metastases were well controlled. These results indicate that radiotherapy may sensitize patients to immunotherapy and increase its efficacy. The results of the combination of durvalumab and tremelimab with radiation were recently presented [77]. Fifteen patients with metastatic BTC who progressed after first-line therapy or refused standard therapy were enrolled in this pilot study. Of 13 patients who received the combination therapy, the DCR was 33%, with 17% PR and 8% CR. Grade 3–4 toxicities were observed in nine out of 15 patients (60%).

In addition to radiotherapy, local ablation is commonly used for treating small liver tumors of up to 5 cm in diameter. Xie C et al. [78] explored the efficacy of the combination of tremelimumab and microwave ablation in 20 patients with advanced BTC. An overall response rate of 12.5% and a disease control rate of 50% were observed. The median PFS, time to progression (TTP), and OS were 3.4 months, 3.3 months and 6.0 months, respectively. The combination treatment was well tolerated, with less than 10% of patients experiencing grade 3–4 toxicity. Profiling of immune cell subsets in peripheral blood revealed an increase in circulating activated (HLA-DR-positive) CD8 + T cells. Selected published trials on the combination of ICIs with immunotherapy, chemotherapy, anti-angiogenics or local therapy in advanced BTC are shown in Table 1.

**Table 1:** Selected published trials on the combination of ICIs with immunotherapy, chemotherapy, anti-angiogenics or local therapy in advanced biliary tract cancer

Author	Country	Trial number	Phase	Treatment Arm(s)	Patients	Line of treatment	ORR (%)	PFS (Months)	OS (Months)	Ref
**ICIs in combination with immunotherapy**										
Ioka T	Japan	NCT01938612	I	Durvalumab (D) + / − Tremelimumab (T)	D (*n* = 42) D + T (*n* = 65)	Second	4.8 in D, 10.8 in D + T	-	8.1 in D, 10.1 in D + T	[45]
Klein O	Australia	CA209-538	II	Nivolumab + Ipilimumab	39	First and Second	23	2.9	5.7	[46]
Kelley RK	US	NCT02703714	II	Pembrolizumab + GM-CSF	27	Second	21	-	-	[49]
**ICIs in combination with chemotherapy**										
Ueno M	Japan	JapicCTI-153098	I	Nivolumab vs Nivolumab + GP chemotherapy	30 vs 30	Second	3 vs 37	1.4 vs 4.2	5.2 vs 15.4	[54]
Sahai V	US	NCT03101566	II	Nivolumab + GP chemotherapy vs Nivolumab + ipilimumab	35 vs 33	First	22.9 vs 3	6.6 vs 3.9	10.6 vs 8.2	[55]
Oh DY	Korea	TOPAZ-1 NCT03875235	III	Durvalumab + GP chemotherapy vs placebo + GP chemotherapy	341 vs 344	First	26.7 vs 18.7	7.2 vs 5.7	12.8 vs 11.5	[58]
Li W	China	NCT03796429	II	Toripalimab + gemcitabine + S1	50	First	30.6	7	15	[60]
Chen X	China	NCT03486678	II	Camrelizumab + GEMOX	38	First	80 in PD-L1 TPS ≥ 1%, 53.8 in TPS < 1%	6.1	11.8	[61]
**ICIs in combination with targeted therapy**										
Arkenau HT	UK	NCT02443324	I	Pembrolizumab + Ramucirumab	26	Second	4	1.6	6.4	[69]
Lin J	China	NCT03892577	Real-world Study	Pembrolizumab or nivolumab + lenvatinib	56	First	30.5	5	11	[70]
Zhang Q	China	ChiCTR2100044476	II	PD-1 inhibitors + Lenvatinib	38	First	42.1	8	17.7	[71]
Zuo B	China	NCT03892577	Real-world Study	PD-1 inhibitors + Lenvatinib	31 (GBC)	First and Second	32.3	5	11.3	[72]
Shi C	China	-	Retrospective study	PD-1 inhibitors + Lenvatinib	74	Second	20.27	4	9.5	[73]
Lulu X	China	-	Retrospective study	Tislelizumab + Lenvatinib	40 (ICC)	Second	17.5	5.83	14.3	[74]
Jian Z	China	NCT03951597	II	Toripalimab + lenvatinib + GEMOX	30	First	80	10	-	[75]
**ICIs in combination with local therapy**										
Hong TS	US	NCT03482102	I	Durvalumab-tremelimumab + radiotherapy	15	Second	20	1.8	-	[91]
Xie C	US	NCT01853618	II	Tremelimumab + microwave ablation	16	Second	12.5	3.4	6	[92]

## *Cancer* Vaccines

Cancer vaccines utilize tumor-specific antigens based on peptides and DCs to stimulate T cells and enhance the antitumor immune response. As tumor-associated antigens (TAAs), Wilms’ tumor protein 1 (WT1) was found in approximately 80% of BTCs, while mucin protein 1 (MUC1) was reported to be overexpressed in 90% of BTCs [79]. A phase I study of WT1 peptide vaccination combined with gemcitabine in 8 patients with advanced BTC reported that the DCR at 2 months was 50% for BTC, and the median OS was 288 days [80]. WT1-specific T cells were detected in 5/8 of the patients after vaccination. Another phase I clinical trial revealed the safety of the MUC1 peptide vaccine in 8 patients with advanced pancreatic and biliary cancers. However, disease progression was noted in 7 patients [81].

Promising results have been obtained by a case report [82]. A personalized multipeptide vaccination composed of seven tumor-associated epitopes was used to treat a patient with metastatic ICC. The vaccine was designed based on whole exome sequencing (WES), whole transcriptome sequencing (WTS) and HLA ligandome analysis of the tumors. After a follow-up period of 41 months, the patient remained clinically healthy, without any radiologically detectable tumors. A phase I trial reported the antitumor effect of vaccination with four peptides in 9 patients with advanced and chemorefractory BTC [83]. Seven of nine patients demonstrated peptide-specific T-cell immune responses, and six of nine had clinical responses. The median PFS and OS were 156 days and 380 days, respectively. Another study by the same group reported the outcome of a three-peptide vaccination that included cell division cycle associated 1 (CDCA1), cadherin 3 (CDH3) and kinesin family member 20A (KIF20A) in nine patients with advanced BTC [84]. The vaccination was well tolerated. All patients had peptide-specific T-cell responses, with 5/9 patients exhibiting stable disease. In a phase II personalized multipeptide vaccine trial, four HLA-matched peptides selected based on preexisting host immunity were administered in 25 advanced BTC patents [85]. Eight (47%) of the 17 patients who completed the first cycle of vaccination demonstrated an induction of T-cell responses to the vaccine peptides. T-cell responses were observed in 4 of 7 patients (57%) at the second cycle of vaccination.

Dendritic cells (DCs) are the most efficient antigen-presenting cells for activating naive T cells and play a role in regulating both innate and adaptive immune responses. Several tumors, including melanoma, pancreatic cancer, prostate cancer, and thyroid cancer, have been treated with DC-based immunotherapy [86–89]. In a retrospective study, 65 patients with nonresectable, recurrent, or metastatic BTC received a DC-based vaccine targeting WT1 and/or MUC1 [90]. The median survival time (MST) was 18.5 months. The DCR and ORR were 29 and 6%, respectively. The combination of chemotherapy and DC-based immunotherapy contributed significantly to the extension of MST. No serious treatment-related AEs were observed.

## Adoptive Cell Therapy

Adoptive cell therapy (ACT) refers to the intravenous transfer of either tumor-resident or peripheral blood-modified immune cells into cancer patients to mediate an antitumor function [91]. Three different types of ACT exist, namely, autologous tumor-infiltrating lymphocyte (TIL) infusion, chimeric antigen receptor T-cell (CAR-T) therapy targeting specific surface tumor antigens and genetically modified T-cell receptor (TCR) T-cell therapy [92].

The presence of TILs in neoplastic tissue is believed to suggest an antitumor immune response by the host and is closely related to clinical prognosis [93]. A patient with metastatic CCA who progressed after multiple rounds of chemotherapy was enrolled in a tumor-infiltrating lymphocyte (TIL)-based ACT protocol for patients with GI cancers [94]. TIL generated from lung metastases contained CD4 + Th1 cells recognizing a mutation in the erbb2 interacting protein (ERBB2IP) expressed by the cancer. The patient received adoptive transfer of TIL followed by interleukin (IL)-2 to enhance T-cell proliferation and function. All target lung and liver lesions markedly shrank after 7 months of follow-up. The patient experienced disease stabilization for approximately 13 months.

Immunotherapy with CAR-T cells has achieved remarkable success in treating hematological malignancies. However, CAR-T therapy for solid tumors faces multiple obstacles, such as trafficking to the tumor sites, the hostile tumor microenvironment, and immune-related adverse events [95]. Feng et al. [96] reported a patient with unresectable/metastatic CCA who was resistant to chemotherapy and radiotherapy and was successively treated with CAR-T cells targeting epidermal growth factor receptor (EGFR) and CD133, respectively. The patient achieved a PR of 8.5 months from the CART-EGFR therapy and 4.5 months from the CART-CD133 treatment. Further investigation is needed on the toxic effects, especially on epidermal/endothelial damage. A phase I study from the same team reported HER2-targeted CAR-T therapy in 11 patients with HER2-positive advanced BTC (*n* = 9) and pancreatic adenocarcinoma (*n* = 2) [97]. One patient obtained a 4.5-month PR, and 5 achieved SD. The median PFS was 4.8 months. CAR-T-cell infusions resulted in mild to moderate adverse events, with the exception of one case of grade 3 acute febrile illness and one transaminase abnormality. Guo Y et al. [98] evaluated the clinical outcomes of ACT using autologous T cells expressing an EGFR-specific CAR for the treatment of EGFR-positive advanced unresectable, relapsed/metastatic BTCs. Ten of the 17 evaluable patients had SD for 2.5 to 15 months, while one achieved CR for 22 months. The median PFS was 4 months. The infusion of CAR T cells was well tolerated; however, the predicted cutaneous and mucosal adverse effects of anti-EGFR therapy were detected.

Currently, there is not enough evidence to support the clinical use of CAR T‑cell treatment for advanced CCA. More clinical trials are required to ensure safe and efficacious clinical application. The safety and efficacy of MUC-1 CAR T cells are under investigation in a Chinese I/II trial for patients with ICC (NCT03633773). Another clinical trial based on EGFR-targeting CAR T-cell therapy for patients with chemotherapy-refractory advanced solid tumors is recruiting (NCT01869166).

## Predictive Markers of Response

Anti-PD-1/PD-L1 immunotherapies have been approved for treating human cancers with considerable clinical effects. Nevertheless, only a minority of patients with these cancers are sensitive to ICI therapies. The establishment of predictive biomarkers for immunotherapy and the understanding of resistance mechanisms in nonresponders is therefore of utmost importance to maximize therapeutic benefits.

### PD-L1

PD-L1 expression assessed through immunohistochemistry is actually considered a logical biomarker for predicting the efficacy of anti-PD-1 or anti-PD-L1 therapy in a number of tumor types [99]. However, there are limited data on the role of PD-L1 in advanced BTC patients. PD-L1 expression in BTC is highly heterogeneous. According to previous studies, PD-L1 expression is found in 9% to 72% of tumors and 46% to 63% of immune cells within the tumor microenvironment [100, 101]. Gani et al. [101] analyzed 54 tumor samples collected from patients undergoing surgery for ICC. Thirty-nine samples (72.2%) were positive for PD-L1 expression on cells in the tumor front. A recent study investigated the genetic and immune biomarker profiles of surgically resected iCCA with singleplex, whole-slide immunohistochemistry [102]. Of note, PD-L1 staining using clone 22C3 was completely absent in the vast majority of samples (*n* = 93/97). Other studies showed higher PD-L1 expression using clones 5H1 or E1L3 [101, 103]. Variation in PD-L1 expression is attributable to the type of clone employed, the variability of thresholds to define PD-L1 positivity, the heterogeneity of the sample, and the small sample size.

A comprehensive meta-analysis of 15 independent studies involving 1,776 BTC patients found that increased PD-L1 expression was associated with poor overall survival but not with disease-free survival or clinical characteristics [104]. Kim H et al. [105] analyzed the prognostic value of PD-L1 expression in 186 patients with advanced BTC. PD-L1 expression was not significantly associated with OS or PFS. Interestingly, in the subgroup analysis, extrahepatic CCA patients negative for PD-L1 expression had better OS and PFS than those positive for PD-L1 expression.

To date, increasing data are available regarding the predictive value of PD-L1 expression in patients treated with ICIs. In a subgroup analysis in the KEYNOTE-158 phase II trial [21], the median PFS in the PD-L1-expressing and nonexpressing subgroups was 1.9 and 2.1 months, respectively; the median OS was 7.2 and 9.3 months, respectively. Recently, nivolumab was investigated as a second-line treatment for 54 advanced BTC patients in a phase II trial [25]. PD-L1-positive patients had a significantly longer median PFS than PD-L1-negative patients (10.4 versus 2.3 months). However, there was no correlation between PD-1-expressing TILs and clinical outcome. The efficacy and safety of toripalimab in combination with gemcitabine and S-1 (GS) in patients with advanced BTCs were investigated in a phase II trial [49]. The biomarker analysis revealed that low PD-L1 expression was strongly associated with poorer PFS, while no significant difference was observed with regard to OS. These results were consistent with several retrospective studies evaluating the anticancer efficacy of lenvatinib plus PD-1 antibody in patients with BTC [60, 61]. Lin J et al. [106] reported that PD-L1-positive tumor cells were associated with improved survival outcomes in both PFS and OS in patients with advanced BTC who received combined treatment with lenvatinib plus pembrolizumab. However, in the phase III TOPAZ-1 trial, the combination of durvalumab and chemotherapy benefited patients with PD-L1-positive and PD-L1-negative tumors, suggesting that PD-L1 status may have limited value in predicting clinical benefit [58]. Considering the limited sample size, the different PD-L1 assays and cutoff values for PD-L1 expression, the role of PD-L1 expression as an immunotherapy biomarker for advanced BTC remains controversial.

### dMMR/MSI-H

DNA mismatch repair (MMR) is a highly conserved repair mechanism in cellular evolution. Inactivation of MMR genes and dysfunction of MMR (dMMR) proteins may lead to microsatellite instability (MSI), which has been recognized as a distinct tumorigenesis pathway [107]. MSI causes the accumulation of mutations, which in turn leads to the formation of neoantigens and the activation of antitumor immune responses.

The incidence of microsatellite instability-high (MSI-H) varies from 0–31.37% in 39 cancer types. The published data concerning the overall incidence of dMMR or MSI-H in BTC are controversial. In a systematic review by Silva [108], the estimated frequencies of dMMR or MSI-H were 5% for gallbladder carcinoma, 10% for ICC and 5–13% for extrahepatic CCAs. In another study, 91 CCAs were stained by immunohistochemistry for MMR genes. MMR deficiency was found in 20 tumors (22%) [109]. Conversely, recent studies from China and Germany revealed that only 1.3% to 2.06% of patients with CCA had dMMR or MSI-H status [110–112].

Studies suggest that dMMR/MSI-H status could serve as a candidate biomarker for predicting ICI response in patients with solid tumors, regardless of cancer type. Based on the results of the KEYNOTE-016, 164, 012, 028, and 158 trials, pembrolizumab was approved by the FDA in 2017 as a second- or higher-line treatment option for patients with unresectable or metastatic dMMR/MSI-H solid tumors, including CCA [113]. This is the first time that one biomarker defined an indication regardless of the primary tumor site. Data from patients with advanced BTC receiving pembrolizumab showed that only one of 24 patients in the KEYNOTE-028 study had MSI-H tumors, and none of 104 patients in the KEYNOTE-158 study had MSI-H tumors [21]. All six of the responding patients in KEYNOTE-158 were microsatellite stable. In the TOPAZ-1 trial [47], only 5 out of 333 (1.5%) patients with evaluable MSI had MSI-H tumors. Consequently, the significant improvement in OS cannot be attributed solely to the efficacy of durvalumab in patients with MSI-H tumors. Due to the limited data available, the value of dMMR/MSI-H in predicting the efficacy of anti-PD-1/PD-L1 immunotherapy in CCA requires further investigation.

### TMB

The tumor mutational burden (TMB) is the total number of somatic nonsynonymous mutations per coding region of a tumor genome. Highly mutated tumors can generate a large number of neoantigens, some of which may increase T-cell reactivity. Several analyses have shown a correlation between high TMB and improved response to treatment with immune checkpoint blockade across several tumor types [114–116]. In the prospective exploratory analysis of the KEYNOTE-158 study, Marabelle A et al. [117] found that TMB-high status was associated with a higher proportion of patients with an objective response than nontTMB-high status among patients who received pembrolizumab monotherapy for previously treated solid tumors. A total of 105 (13%) of the 805 patients had a TMB-high status. Most of them had lung and cervical cancer, and none had BTC. The proportions of patients with an objective response in the TMB-high and non-TMB-high groups were 29% and 6%, respectively. Based on these findings, anti-PD-1 therapy was approved by the FDA in 2020 for solid tumors with a TMB of 10 or more mutations per megabase.

Data on the prevalence of TMB-high BTC are limited. Through a comparative genomic profiling analysis of 309 tissues from patients with BTC, TMB-high (TMB ≥ 20 mut/Mb) was found in only 2.9% of cases [118]. A retrospective cohort study using a real-world dataset evaluated the prevalence of TMB-high (TMB ≥ 10 mut/Mb) among 2589 patients with 10 tumor types [119]. The prevalence of TMB-high varies widely depending on tumor type. The prevalence of TMB-high was highest in patients with SCLC (40.0%), NETs (29.3%), and cervical cancer (14.9%), whereas it was lowest in patients with mesothelioma (1.2%), thyroid cancer (2.7%), and biliary cancer (4.0%). In addition, the median TMB varied considerably among the different tumors, with a range from 1.7 mut/Mb to 8.7 mut/Mb. Therefore, the optimal TMB threshold to identify patients for immunotherapy needs to be defined based on the tumor type. Moreover, the frequency of TMB-high differed significantly between BTC subtypes. TMB is significantly higher in eCCA (18%) and GBC (22%) than in iCCA (13%) [120].

Few studies have explored the value of TMB for predicting the response to immunotherapy in patients with BTC. Moreover, the results were inconsistent and conflicting. A series of 24 patients with relapsed or advanced BTC was examined with next-generation sequencing (NGS) [121]. Three patients with TMB-high tumors were treated with nivolumab and chemotherapy. All these patients achieved complete response or partial response. In a retrospective cohort study of 1678 patients with MSS tumors from 16 cancer types who were treated with ICI therapy [122], response rates were generally higher with a high TMB (10 mutations per megabase). However, of 53 patients with hepatobiliary tumors, four (7.5%) patients had a high TMB; none responded to immunotherapy. Zhou C et al. [123] evaluated the value of WES-derived TMB in 309 patients who had received ICI therapy across 5 different cancers. When a fixed number of 10 mutations/Mb was used as the threshold criterion to stratify patients, only 4 of 62 (6.45%) patients with CCA had a TMB-high status, which prevented a meaningful statistical comparison. When the top tertile TMB value was used as the divider, unexpectedly, the TMB-high group had a lower OS than the TMB-low group, although this difference did not achieve statistical significance. Therefore, a strong correlation between TMB-high and ICI benefit in patients with CCA could not be established in this cohort. In the first trial of lenvatinib plus PD-1 inhibitors for the first-line treatment of BTC, Zhang Q et al. [59] also reported that patients with lower TMB had a significantly better ORR and longer OS than patients with higher TMB. In contrast, a real-world retrospective study revealed a prolonged PFS in the TMB-high group among 74 patients with advanced BTC treated with lenvatinib and PD-1 inhibitors [61]. Exploratory analysis of biomarkers in a phase II study that enrolled 50 patients who received toripalimab combined with gemcitabine and S-1 indicated that TMB was not associated with clinical response [49], which is consistent with another recently published study in China [124]. Overall, due to the lack of standardization of tissue TMB calculation, robust predictive cutoffs and the complexity of immune response and resistance, further studies are needed to elucidate the role of TMB in predicting the response to ICIs in BTC patients.

### DNA Damage Repair

The development and progression of CCA have been associated with increased DNA damage and genomic instability. The DNA damage response (DDR) is a multicomponent, structured network that has developed to repair damaged DNA, maintain genome fidelity and avoid the accumulation of mutations [125]. DDR gene aberrations impair DNA damage repair processes and drive genomic instability, which may increase tumor immunogenicity and induce a better response to immunotherapy [126]. Next-generation profiling of CCA revealed the presence of alterations in DDR-related genes, including BRCA1/2, PARP, ATM, ATR, BAP1, ARID1A, RAD51, MLH1, TP53, PALB2, PTEN, FANC, NBN, EMSY and MRE11 [127]. The most frequently altered DDR genes were ATM (5%) and BRCA1/2 (4.8%) [128]. Approximately 30% of BTC patients have DDR gene mutations, whereas the rate of BRCA mutations fluctuates between 1 and 7% [129–131].

Spizzo G et al. [132] analyzed tumor samples from 1292 patients with BTC using NGS. BRCA mutations were detected in 3.6% (*n* = 46) of 1292 BTC samples. The results regarding the association of BRCA1/2 mutations with the predictive biomarkers of response to ICIs showed that the median TMB level was significantly higher in BRCA-mutant BTC than in BRCA-WT BTC. Moreover, MSI-H/dMMR was observed more frequently in patients with BRCA-mutant BTC. Therefore, immune checkpoint inhibition may benefit patients with BRCA-mutated BTC. Nonetheless, regarding the methods for testing and defining DDR alterations in BTC, no consensus has been reached thus far. In addition, due to the low incidence of single DDR gene mutations, most small-sample studies have been unable to establish a direct association between DDR gene mutations and the response to ICIs in BTC patients. Further studies are warranted in this direction.

### Genetic Alterations

A comprehensive understanding of the genetic characteristics is essential for identifying biomarkers that predict the response to immunotherapy for BTC patients. Chen X et al. [133] assessed the genomic profiles of 98 patients with advanced BTC (CCA, *n* = 62; GBC, *n* = 36). The most commonly mutated gene in both CCA (55%) and GBC (72%) was TP53. CCA exhibited a high incidence of KRAS-TP53 comutations, which was associated with a favorable response to camrelizumab (ORR = 75%), while CCA with a single KRAS mutation had a poor immunotherapy outcome. Furthermore, eleven mutated genes with the most powerful prognostic role were selected to develop a genetic signature. The signature wild-type subtype responded better to camrelizumab than its mutated subtype. ERBB2 and ERBB3 mutations have been implicated in a variety of cancers. Li M et al. [134] performed WES to identify novel somatic mutations in 157 patients with GBC. ERBB2 and ERBB3 mutations were observed in 7%-8% of patients. These mutations upregulated PD-L1 expression in GBC cells and effectively suppressed normal T-cell-mediated cytotoxicity in vitro. PD-L1 blockade can enhance the efficacy of anti-ERBB therapy in GBC cells with ERBB2/ERBB3 mutations.

Recently, a comprehensive genomic characterization of a large Western cohort (*n* = 189) of extrahepatic CCA (eCCA) was performed by Montal R et al. [135]. Four molecular classes (metabolic, proliferation, mesenchymal and immune) were identified based on an integrative molecular analysis. The eCCA immune class had overexpression of PD-1/PD-L1 and displayed marked lymphocytic infiltration. Therefore, the molecular features in the eCCA immune class may define a population that could benefit from ICIs. Yoon JG et al. [136] identified the molecular features of treatment responses to PD-1/PD-L1 blockade therapy in 48 BTC patients. A high intratumoral TIL level at the tumor center was the most important determinant of clinical benefit. KRAS alterations and chromosomal instability (CIN) were found to be associated with a lower clinical benefit rate (CBR) to PD-1/PD-L1 blockade and a shorter PFS after immunotherapy. Patient stratification based on KRAS alterations, CIN status and PD-L1 expression may improve the clinical benefit of immunotherapy in BTC patients.

## Discussion

Biliary tract cancers are heterogeneous neoplasms of poor prognosis with specific anatomical, molecular and biological characteristics. Advanced BTC remains a difficult-to-treat malignancy. Most patients are deprived of curative options due to the high heterogeneity and complex tumor microenvironment (TME). Consequently, the development of more effective and personalized treatment options is a great unmet need. In recent years, immunotherapy has brought revolutionary changes in the field of cancer treatment. The role of immunotherapy in advanced BTC is currently being investigated (Table 2). The limited response to ICI monotherapy in unselected patients and the immune-suppressed nature of the biliary TME emphasize the need for the development of combinational therapeutic approaches along with predictive biomarkers.

**Table 2:** Ongoing clinical trials of immunotherapy in BTC

Trial identifier	Agent	Target/mechanism	Cancer type	Estimated enrollment	Clinical phase	Curntry/region	Status
**ICIs monotherapies**							
NCT02829918	Nivolumab	Anti-PD-1	Advanced Refractory BTC, 2nd line	54	II	USA	Active, not recruiting
NCT02628067	Pembrolizumab	Anti-PD-1	Advanced Solid Tumors, including BTC, 2nd line	1609	II	International Multicentre	Recruiting
NCT03695952	Nivolumab or pembrolizumab	Anti-PD-1	HCC or BTC, 1st line or 2nd line	100	Prospective observational cohort	Korea	Recruiting
NCT02829918	Nivolumab	Anti-PD-1	Advanced Refractory BTC, 2nd line	54	II	USA	Active, not recruiting
NCT02091141	Atezolizumab	Anti-PD-L1	Advanced, refractory solid tumours, including BTC with high TMB, 1st line	765	IIa	USA	Active, not recruiting
**ICIs in combination with immunotherapy**							
NCT05653180	IBI310 + Sintilimab	Anti-CTLA4, anti-PD1	Advanced BTC, 2nd line	20	Ib/II	China	Recruiting
NCT05540483	Disitamab Vedotin + Zimberelizumab	Anti-HER2, anti-PD1	HER2-overexpressed, Previously Treated Unresectable BTC, 2nd line	31	II	China	Recruiting
NCT04066491	Gemcitabine-Cisplatin + / − M7824	Chemotherapy, Anti-TGF-ß-PD-L1	Locally Advanced or Metastatic BTC, 1st line	309	II/III	International Multicentre	Active, not recruiting
NCT04298008	AZD6738 + Durvalumab	Anti-ATR kinase, Anti-PD-L1	Refractory BTC, 2nd line	26	II	Korea	Recruiting
NCT02834013	Nivolumab + Ipilimumab	Anti-PD-1, anti-CTLA-4	Advanced, refractory solid tumors, including BTC, 2nd line	818	II	USA	Active, not recruiting
NCT03668119	Nivolumab + / − Ipilimumab	Anti-PD-1, anti-CTLA-4	Advanced or Metastatic Solid Tumors with high TMB, including CCA, 2nd line	212	II	International Multicentre	Active, not recruiting
**ICIs in combination with chemotherapy**							
NCT03796429	Toripalimab + gemcitabine + S1	Anti-PD-1, chemotherapy	Advanced BTC, 1st line	40	II	China	Recruiting
NCT04172402	Nivolumab + Gemcitabine + TS1	Anti-PD-1, chemotherapy	Advanced BTC, 1st line	48	II	Taiwan, China	Active, not recruiting
NCT05771480	Durvalumab + gemcitabine-based chemotherapy	Anti-PD-L1, chemotherapy	Advanced BTC, 1st line	160	IIIb	International Multicentre	Not yet recruiting
NCT03785873	Nivolumab + nanoliposomal-irinotecan + 5-fluorouracil + and leucovorin	Anti-PD-1, chemotherapy	Advanced BTC, ^2^nd line	34	Ib/II	USA	Active, not recruiting
NCT03260712	Pembrolizumab + Gemcitabine + Cisplatin	Anti-PD-1, chemotherapy	Advanced or Metastatic BTC, 1st line	50	II	Germany, Spain, UK	Active, not recruiting
NCT04003636	Pembrolizumab + Gemcitabine + Cisplatin vs Gemcitabine + Cisplatin	Anti-PD-1, chemotherapy	Advanced and/or unresectable BTC, 1st line	1069	III	International Multicentre	Active, not recruiting
NCT03478488	Gemcitabine + Oxaliplatin ± KN035	Anti-PD-L1, chemotherapy	Previously untreated locally advanced or metastatic BTC, 1st line	480	III	China	Recruiting
NCT04191343	Toripalimab + GEMOX	Anti-PD-1, chemotherapy	Advanced BTC, 1st line	20	II	China	Recruiting
NCT03473574	Durvalumab + Tremelimumab + Gemcitabine ± Cisplatin vs Gemcitabine + Cisplatin	Anti-PD-L1, anti-CTLA-4, chemotherapy	Untreated BTC, 1st line	128	II	Germany	Active, not recruiting
NCT03046862	Durvalumab + Tremelimumab + Gemcitabine + Cisplatin	Anti-PD-L1, anti-CTLA-4, chemotherapy	Chemotherapy-naïve, Unresectable or recurrent BTC, 1st line	31	II	Korea	Active, not recruiting
NCT03704480	Durvalumab + Tremelimumab ± Paclitaxel	Anti-PD-L1, anti-CTLA-4, chemotherapy	Advanced BTC, 2nd line	106	II	France	Recruiting
NCT03257761	Durvalumab + Guadecitabine	Anti-PD-L1, chemotherapy	Unresectable, refractory HCC, PDAC, or BTC excluding ampullary, 2nd line	55	Ib	USA	Active, not recruiting
NCT04413734	Triprilumab + Gemcitabine + Cisplatin	Anti-PD-1, chemotherapy	Unresectable ICC, 1st line	120	II	China	Recruiting
NCT04308174	Gemcitabine + Cisplatin ± Durvalumab	Anti-PD-L1, chemotherapy	Resectable BTC, neoadjuvant	45	II	Korea	Active, not recruiting
**ICIs in combination with targeted therapy**							
NCT05056116	Toripalimab + Surufatinib	Anti-PD-1, TKI	Advanced (unresectable) or metastatic BTC, 2nd line	30	II	China	Recruiting
NCT04550624	Pembrolizumab + Lenvatinib	Anti-PD-1, TKI	Advanced BTC, 2nd line	40	II	China	Recruiting
NCT04211168	Toripalimab + Lenvatinib	Anti-PD-1, TKI	Advanced BTC, 2nd line	44	II	China	Recruiting
NCT04781192	Durvalumab + Regorafenib	Anti-PD-L1, TKI	Chemo Refractory Advanced BTC, 2nd line	40	I/II	USA	Recruiting
NCT05222971	Olaparib ± Durvalumab	Anti-PARP, anti-PD-L1	DDR Gene Mutated Advanced BTC, 2nd line	62	II	Korea	Recruiting
NCT04010071	Axitinib + Toripalimab	Anti-VEGFR, anti-PD-1	Hepatobiliary malignant tumors, 2nd line	60	II	China	Recruiting
NCT03201458	Atezolizumab ± Cobimetinib	Anti-PD-L1, anti-MEK	Unresectable, refractory BTC, 2nd line	86	II	USA	Active, not recruiting
NCT03639935	Nivolumab + Rucaparib	Anti-PD-1, anti-PARP	Advanced or Metastatic BTC, 2nd line	32	II	USA	Active, not recruiting
NCT03991832	Durvalumab + Olaparib	Anti-PD-L1, anti-PARP	Selected solid tumours, including BTC, with IDH mutations, 1st line	58	II	Canada	Recruiting
NCT03829436	Nivolumab + TPST-1120	Anti-PD-1, anti-PARP	Advanced solid tumours, including CCA, 1st line	138	I	USA	Active, not recruiting
NCT03095781	Pembrolizumab + XL888	Anti-PD-1, anti-HSP90	Advanced, refractory GI cancers, including CCA, 2nd line	49	Ib	USA	Active, not recruiting
NCT03797326	Pembrolizumab + Lenvatinib	Anti-PD-1, TKI	Advanced, refractory solid tumours, including BTC, 2nd line	590	II	International Multicentre	Active, not recruiting
NCT03475953	Avelumab + Regorafenib	Anti-PD-L1, TKI	Advanced, unresectable or metastatic Solid Tumors, including BTC, not MSI-H or deficient-MMR, 2nd line	747	II	France	Recruiting
NCT04057365	Nivolumab + DKN-01	Anti-PD-1, anti-DKK1	Advanced BTC, 2nd line	30	II	USA	Recruiting
NCT03257761	Durvalumab + Guadecitabine	Anti-PD-L1, anti-DNMT	Advanced Liver, Pancreatic, Bile Duct, or Gallbladder Cancer, 2nd line	55	Ib	USA	Active, not recruiting
NCT04454905	Camrelizumab + Apatinib	Anti-PD-1, TKI	Advanced ICC, 1st line	50	II	China	Recruiting
NCT04306367	Pembrolizumab + Olaparib	Anti-PD-1, anti-PARP	Advanced Cholangiocarcinoma, 2nd line	13	II	USA	Active, not recruiting
NCT04298021	Durvalumab + Ceralasertib vs Olaparib + Ceralasertib	Anti-PD-L1, anti-ATR, anti-PARP	Advanced BTC, 2nd line	74	II	Korea	Recruiting
NCT04301778	Durvalumab + SNDX-6532	Anti-PD-L1, anti- CSF-1R	Unresectable ICC post chemoembolization or radioembolization, 2nd line	5	II	USA	Active, not recruiting
**ICIs in combination with targeted therapy and chemotherapy**							
NCT05742750	Camrelizumab + Apatinib + Gemcitabine + Cisplatin	Anti-PD-1, TKI, chemotherapy	Inoperable/Metastatic BTC, 1st line	48	Ib/II	China	Not yet recruiting
NCT05451290	Camrelizumab + Apatinib + GEMOX	Anti-PD-1, TKI, chemotherapy	Locally Advanced BTC, 1st line	30	II	China	Not yet recruiting
NCT05052099	Atezolizumab + Bevacizumab + mFOLFOX6	Anti-PD-L1, anti-VEGF, chemotherapy	Advanced BTC, 2nd line	35	Ib/II	Germany, Spain	Recruiting
NCT04300959	Sintilimab + Anlotinib + Gemcitabine + Cisplatin	Anti-PD-1, TKI, chemotherapy	Unresectable or Metastatic BTC, 1st line	80	II	China	Recruiting
NCT04677504	Atezolizumab + / − Bevacizumab + Gemcitabine + Cisplatin	Anti-PD-L1, anti-VEGF, chemotherapy	Untreated, Advanced BTC, 1st line	162	II	International Multicentre	Active, not recruiting
NCT05749900	Nivolumab + Trastuzumab + Gemcitabine + Cisplatin	Anti-PD-1, anti-HER2, chemotherapy	Advanced HER2- Positive BTC, 1st line	44	Ib/II	Korea	Not yet recruiting
NCT05410197	Envofolimab + Lenvatinib + Gemcitabine + Cisplatin	Anti-PD-L1, TKI, chemotherapy	Advanced BTC, 1st line	43	II	China	Recruiting
NCT04720131	Camrelizumab + Apatinib + Capecitabine	Anti-PD-1, TKI, chemotherapy	Advanced Unresectable BTC, 1st line	39	II	China	Recruiting
NCT05156788	Tislelizumab + Lenvatinib + Gemox	Anti-PD-1, TKI, chemotherapy	Potentially resectable, locally advanced BTC, 1st line	40	II	China	Recruiting
NCT04984980	Gemcitabine + Oxaliplatin + Sintilimab + Bevacizumab	Anti-PD-L1, anti-VEGF, chemotherapy	Initially Unresectable BTC, 1st line	37	II	China	Active, not recruiting
NCT05668884	GEMOX + Donafenib + Tislelizumab	Anti-PD-1, TKI, chemotherapy	Advanced BTC, 1st line	35	II	China	Recruiting
NCT04669496	Toripalimab + Lenvatinib + GEMOX	Anti-PD-1, TKI, chemotherapy	Resectable ICC with high-risk recurrence factors, neoadjuvant	178	II/III	China	Recruiting
**ICIs in combination with local therapy**							
NCT03482102	Durvalumab + Tremelimumab + radiation	Anti-PD-L1, anti-CTLA-4, local	Locally advanced, unresectable or metastatic HCC or BTC, 2nd line	70	II	USA	Recruiting
NCT03898895	Camrelizumab + radiation	Anti-PD-1, local	Unresectable BTC, 1st line	36	II	China	Recruiting
NCT04238637	Durvalumab + Tremelimumab + Y90 SIRT	Anti-PD-L1, anti-CTLA-4, local	Intrahepatic BTC, 2nd line	50	II	Germany	Recruiting
NCT02866383	Nivolumab + radiation + / − Ipilimumab	Anti-PD-1, local + / − anti-CTLA-4	Metastatic Pancreatic Cancer or BTC, 2nd line	160	II	Denmark	Active, not recruiting
NCT04708067	Hypofractionated Radiation Therapy-M7824	Local, anti-TGF-ß-PD-L1	Advanced ICC, 2nd line	15	I	USA	Recruiting
NCT05781074	Cryoablation + Sintilimab + lenvatinib	Local, anti-PD-1, TKI	Advanced BTC, 2nd line	25	II	China	Recruiting
NCT04217954	HAIC(Oxaliplatin + 5-FU) + Bevacizumab + Toripalimab	Local, anti-VEGF, anti-PD-1	Advanced BTC, 1st line	32	II	China	Recruiting
NCT03937830	Durvalumab + Bevacizumab + Tremelimumab + TACE	Local, anti-VEGF, anti-PD-L1, anti-CTLA-4	HCC or BTC, 2nd line	39	II	USA	Recruiting
NCT04299581	Cryoablation + Camrelizumab	Local, anti-PD-1	Advanced ICC, 2nd line	25	II	China	Recruiting
NCT04068194	Peposertib + Avelumab + Radiation	Local, anti-DNA-PK, anti-PD-L1	Advanced, Metastatic Solid Tumors and Hepatobiliary Malignancies, 2nd line	39	I/II	USA	Recruiting
**Adoptive cell therapy**							
NCT03633773	MUC-1 CART cell	ACT	ICC, 1st line	9	I/II	China	Recruiting
NCT03801083	Tumor Infiltrating Lymphocytes (TIL)	ACT	Locally Advanced, Recurrent, or Metastatic, 1st and 2nd line	59	II	USA	Recruiting
NCT02482454	cytokine-induced killer cells (CIK) + radiofrequency ablation (RFA)	ACT	Unresected CCA, without extrahepatic metastasis, 1st line	50	III	China	Recruiting

Based on current evidence from diverse clinical studies, ICIs in combination with immunotherapy, chemotherapy, anti-angiogenics, PARP inhibitors or local therapy could increase the sensitivity of BTC to immune therapies. However, the interpretation of available data is challenged by small sample sizes, single-arm designs, and inherent clinical and molecular heterogeneity. TOPAZ-1 is the first phase 3 trial to demonstrate that the addition of immunotherapy to standard chemotherapy can increase survival in biliary tract cancer, importantly, without inducing any new serious side effects [47]. In the latest NCCN guidelines for hepatobiliary cancers, durvalumab plus gemcitabine and cisplatin were adjusted as preferred regimens, representing a milestone for the management of advanced BTC. Recently, the combination of a PD-1 inhibitor with lenvatinib as first-line or second-line treatment for advanced BTC has shown remarkable results in several phase II and retrospective trials, with objective response rates ranging from 10% to 42.1% and median OS ranging from 8.6 to 17.7 months [58–62]. Further randomized phase III studies are still needed to determine their advantages over standard treatments. Given the genetic heterogeneity of advanced BTC, it is essential to obtain genomic profiles from all patients to guide therapeutic options. Because of the interaction between oncogenic and immunologic pathways, combining molecular and immunotherapies may be a promising approach for BTC. For instance, mutations in HER2/HER3 have been linked to PD-L1-mediated immune escape [137]. Mutations in oncogenes such as BRAF and KRAS are more common in BTC with PD-L1 positivity [138]. Combining PD-1 antibodies with targeted agents is now FDA approved for the treatment of advanced melanoma (BRAF/MEK inhibition) and gastric cancer (anti-HER2) [139, 140].

Revealing robust biomarkers to select patients who could benefit from immunotherapies is an important future research direction. Biomarker development in BTC is complicated by the heterogeneity of the tumor and its microenvironment, as well as the scarcity of tumor tissue obtainable for clinical diagnostics or research [141]. The majority of biomarkers of response and resistance to checkpoint blockade have focused on tumor-intrinsic or immune-specific markers, such as PD-L1, TMB-high, and MSI-H/dMMR. The role of PD-L1 as a biomarker in BTC has been mainly studied retrospectively in small phase I/II studies. Randomized studies are required to evaluate the value of PD-L1 IHC. High TMBs (> 10 or > 20 Mut/Mb) are rare in patients with BTCs, and there is no consensus on the optimal TMB cutoff for BTC. Reports about the value of TMB for predicting the response to immunotherapy are inconsistent. MSI-H/dMMR status is exceedingly rare, accounting for only 1% ~ 2% of patients with BTC. Therefore, novel biomarkers are required to identify BTC patients who will benefit from immunotherapy. Other promising biomarkers are currently being investigated, such as the tumor microenvironment and DNA damage repair [68]. Moreover, it seems unlikely that a single biomarker will be adequate to identify patients for immunotherapy. Combinatorial biomarker strategies with the capacity to dynamically capture and integrate various TME parameters will be required [142]. In addition, due to the scarcity of adequate tumor tissue for molecular profiling, liquid biopsy techniques, such as ctDNA in blood or bile, may have particular advantages [143, 144]. In recent years, the field of radiomics has just begun to emerge in medical oncology. Radiomics enables the extraction of a vast quantity of data, including biomolecular and genetic information, by analyzing the texture of radiological images [145]. Further studies on the prognostic value of radiomics as an imaging biomarker in BTC are needed.

Early clinical trials of therapeutic cancer vaccination and adoptive cell therapy have shown encouraging clinical results. However, there is still a long way to go via the validation of therapeutic efficacy and exploration of strategies to increase the efficacy. The strategy of improving clinical efficacy includes discovering new antigens to customize more personalized immunotherapy. In addition, deep integrative genomic analysis can identify significant and potentially immunogenic genetic mutations. For example, Pandey A et al. [146] analyzed 167 gallbladder cancers and identified ELF3 as a significantly mutated gene. Several neoantigens produced by ELF3 mutations are able to activate CD8 + T cells, suggesting that they may be potential cancer vaccine candidates. Available evidence indicates that the tumor microenvironment plays an important role in the progression of BTC [147, 148]. Tumor and immune cells, along with vasculature, extracellular matrix, and signaling molecules, regulate immune responses and influence the effectiveness of immunotherapy. BTC is characterized by a prominent desmoplastic TME with immunosuppressive innate immune cells. The modulation of the crosstalk between BTC and TME by targeting different components of the TME in combination with cytotoxic treatments or ICIs represents an attractive therapeutic prospective [149].

## **Key References**

- Vogel A, Bridgewater J, Edeline J, Kelley RK, Klümpen HJ, Malka D, et al. Biliary tract cancer: ESMO Clinical Practice Guideline for diagnosis, treatment and follow-up. Ann Oncol. 2023;34(2):127-40.
  - This article provides key recommendations for managing biliary tract cancer, including clinical and pathological diagnosis, staging and risk assessment, treatment and follow-up.
- Valle JW, Kelley RK, Nervi B, Oh DY, Zhu AX. Biliary tract cancer. Lancet. 2021;397(10272):428-44.
  - This article presents new advances in the etiology, diagnosis, molecular classification and treatment of biliary tract cancer.
- Moris D, Palta M, Kim C, Allen PJ, Morse MA, Lidsky ME. Advances in the treatment of intrahepatic cholangiocarcinoma: An overview of the current and future therapeutic landscape for clinicians. CA Cancer J Clin. 2022.
  - This article focuses on current and future ICC treatment strategies, with detailed discussion of surgical and local interventions as well as systemic therapies.
- Lamarca A, Palmer DH, Wasan HS, Ross PJ, Ma YT, Arora A, et al. Second-line FOLFOX chemotherapy versus active symptom control for advanced biliary tract cancer (ABC-06): a phase 3, open-label, randomised, controlled trial. Lancet Oncol. 2021;22(5):690-701.
  - The first randomised phase 3 clinical trial to explore the role of second-line chemotherapy in advanced biliary tract cancer. It showes that FOLFOX can improve median overall survival in patients with advanced biliary tract cancer after progression following cisplatin and gemcitabine.
- Oh D-Y, Ruth He A, Qin S, Chen L-T, Okusaka T, Vogel A, et al. Durvalumab plus Gemcitabine and Cisplatin in Advanced Biliary Tract Cancer. NEJM Evidence. 2022;1(8):1-11.
  - A randomised, double-blind, global phase 3 trial to evaluate the efficacy and safety of durvalumab in combination with gemcitabine and cisplatin versus placebo in combination with gemcitabine and cisplatin as a first-line treatment for patients with advanced biliary tract cancer.
- Li W, Wang Y, Yu Y, Li Q, Wang Y, Zhang C, et al. Toripalimab in advanced biliary tract cancer. Innovation (Camb). 2022;3(4):100255.
  - A phase II, open-label, single-arm, single-centre clinical trial demonstrates the efficacy and safety of toripalimab in combination with GS as first-line treatment for patients with advanced biliary tract cancer.
- Carapeto F, Bozorgui B, Shroff RT, Chagani S, Solis Soto L, Foo WC, et al. The  immunogenomic landscape of resected intrahepatic cholangiocarcinoma. Hepatology. 2022;75(2):297-308.
  - This is the first extensive immune marker immunohistochemistry panel in CCA, providing a solid foundation for the rational improvement and personalization of immunotherapeutic strategies.
- Valero C, Lee M, Hoen D, Zehir A, Berger MF, Seshan VE, et al. Response Rates to Anti-PD-1 Immunotherapy in Microsatellite-Stable Solid Tumors With 10 or More Mutations per Megabase. JAMA Oncol. 2021;7(5):739-43.
  - The data from this cohort study demonstrates that immune checkpoint inhibitor therapy for tumours with 10 or more mutations per megabase of TMB generally has a higher response rate across multiple cancer types. However, the predictive value of a single universal threshold for TMB -high tumours is limited because it varies widely across cancer types.
- Yoon JG, Kim MH, Jang M, Kim H, Hwang HK, Kang CM, et al. Molecular Characterization of Biliary Tract Cancer Predicts Chemotherapy and Programmed Death 1/Programmed Death-Ligand 1 Blockade Responses. Hepatology. 2021;74(4):1914-31.
  - This article uses a clinical sequencing platform to predict the molecular characteristics of chemotherapy and immunotherapy responses in advanced biliary tract cancer.
- Martin-Serrano MA, Kepecs B, Torres-Martin M, Bramel ER, Haber PK, Merritt E, et al. Novel microenvironment-based classification of intrahepatic cholangiocarcinoma with therapeutic implications. Gut. 2022.
  - This article describes a comprehensive TME-based iCCA composite stratification and calls it stroma, tumour, immune microenvironment based or STIM.
